# The presence of meniscal lesions is a strong predictor of neuropathic pain in symptomatic knee osteoarthritis: a cross-sectional pilot study

**DOI:** 10.1186/s13075-014-0507-z

**Published:** 2014-12-14

**Authors:** Camille Roubille, Jean-Pierre Raynauld, François Abram, Patrice Paiement, Marc Dorais, Philippe Delorme, Louis Bessette, André D Beaulieu, Johanne Martel-Pelletier, Jean-Pierre Pelletier

**Affiliations:** Osteoarthritis Research Unit, University of Montreal Hospital Research Centre (CRCHUM), 900 rue Saint-Denis, Pavillon R, Suite R11.412, Montreal, QC Canada H2X 0A9; Medical Imaging Research & Development, ArthroLab Inc, 1871 rue Sherbrooke Est, Montreal, QC Canada H2K 1B6; StatSciences Inc, 60 rue Sylvio Leduc, Notre-Dame de l’Île-Perrot, QC Canada J7V 7P2; Groupe de Recherche en Rhumatologie et Maladies Osseuses, 1200 Avenue de Germain-des-Prés, Suite 100, Sainte-Foy, QC Canada G1V 3M7; Centre de rhumatologie St-Louis, 3165 chemin St-Louis, Suite 360, Sainte-Foy, QC G1W 4R4 Canada

## Abstract

**Introduction:**

Pain in osteoarthritis (OA) has been classically attributed to joint structural damage. Disparity between the degree of radiographic structural damage and the severity of symptoms implies that factors other than the joint pathology itself contribute to the pain. Peripheral and central sensitization have been suggested as two of the underlying mechanisms that contribute to pain in OA. The aim of this study was to explore in symptomatic knee OA patients, the structural changes assessed by magnetic resonance imaging (MRI) that could be used as markers of neuropathic pain (NP).

**Methods:**

This cross-sectional observational pilot study included 50 knee OA patients with moderate to severe pain (VAS ≥40) in the target knee. The presence of NP was determined based on the PainDETECT questionnaire. Among the 50 patients included, 25 had PainDETECT score ≤12 (unlikely NP), 9 had PainDETECT score between 13 and 18 (uncertain NP) and 16 had PainDETECT score ≥19 (likely NP). WOMAC, PainDETECT, and VAS pain scores as well as knee MRI were assessed.

**Results:**

Data showed no significant difference in demographic characteristics between the three groups. However, a positive and statistically significant association was found between the WOMAC pain (*P* <0.001), function (*P* <0.001), stiffness (*P* = 0.007) and total (*P* <0.001) scores as well as higher VAS pain score (*P* = 0.023), and PainDETECT scores. Although no difference was found in the cartilage volume between groups, the presence of meniscal extrusion in both medial (*P* = 0.006) and lateral (*P* = 0.023) compartments, and presence of meniscal tears in the lateral compartment (*P* = 0.011), were significantly associated with increasing PainDETECT score. Moreover, the presence of bone marrow lesions in the lateral plateau and the extent of the synovial membrane thickness in the lateral recess were associated with increasing PainDETECT scores (*P* = 0.032, *P* = 0.027, respectively).

**Conclusions:**

In this study, meniscal lesions, particularly extrusion, were found to be among the strongest risk factors for NP in knee OA patients.

**Trial registration:**

ClinicalTrials.gov NCT01733277. Registered 16 November 2012.

## Introduction

Pain in osteoarthritis (OA) has been classically attributed to joint structural damage, and nearly all therapeutic strategies have been aimed at treating the pain derived from the joint. However, disparity between the degree of radiographic structural damage and the severity of symptoms, such as pain and functional limitations in OA patients, implies that factors other than the joint pathology itself, also contribute to the pain. Peripheral and central sensitizations have been suggested as two of the underlying mechanisms of pain in OA. Indeed, OA patients have been found to experience both nociceptive and neuropathic pain (NP) to varying degrees [1-5]. The peripheral nociceptors may be sensitized by, for example, inflamed synovium and damaged subchondral bone [5]. Continuous and intense nociceptive input from the OA knee joint may drive central sensitization, which may arise from chronic nociceptor stimulation and subsequent modification of central pain-transmitting neurons, and may be clinically associated with NP qualities [1,2]. The diagnosis of NP is clinical and based on medical history, physical examination, and ancillary tests [6]. One such test, the PainDETECT, is a patient-report questionnaire extensively validated for the diagnosis of NP in various chronic pain conditions [7-11]. Higher scores suggest the likelihood of NP while lower scores are suggestive of a nociceptive pain. Hence, an NP component is unlikely if the score is ≤12, uncertain if the score is 13 to 18, and likely if the score is ≥19 [7].

This cross-sectional study thus aimed at investigating, in knee OA patients with moderate to severe pain (visual analog scale (VAS) ≥40 mm), the structural changes assessed by magnetic resonance imaging (MRI) that could be used as markers of uncertain (PainDETECT >12) or likely (PainDETECT ≥19) NP.

## Methods

### Study population and design

This study is a multicentre, cross-sectional, single-blinded observational pilot study. The presence of NP was determined based on use of the PainDETECT questionnaire (score 0 to 38) [7-11]. The classification method defines NP as unlikely (score ≤12), uncertain (score 13 to 18), or likely (≥19). In this study, a total of 50 knee OA patients with moderate to severe pain (VAS ≥40) in the most painful knee were enrolled, 25 having PainDETECT score ≤12, 9 having PainDETECT score between 13 and 18, and 16 having PainDETECT score ≥19. Since this is a pilot study, the number of patients was arbitrarily determined at 50, which represents 25 patients with unlikely NP and 25 patients with uncertain or likely NP, a number deemed sufficient to identify the knee OA structural changes that could be preferentially associated with NP.

Subjects aged 40 years and older, followed in ambulatory clinics, with a diagnosis of primary knee OA according to the American College of Rheumatology criteria, of Kellgren-Lawrence (KL) radiological grades 2 and 3, and being symptomatic for at least 1 month out of the 3 months preceding the study, and VAS pain score while walking on a flat surface ≥40 mm, were eligible to be included in the study.

Subjects were excluded from the study if they met any of the following criteria at the beginning of, or during, the study: other bone or articular diseases (antecedents and/or current signs), isolated knee OA in the lateral compartment only defined by joint space loss, surgery on the target knee, comorbidities that restrict knee function, having taken any investigational drug within 30 days or 5 half-lives (whichever is greater) prior to entering the study, inability to give informed consent, meeting any contra-indication related to MRI, having taken either corticosteroids (oral, injectable or intra-articular injection of the target knee during the 12 weeks preceding the study) or intra-articular injections of hyaluronic acid in the target knee during the 26 weeks preceding the study.

This study, registered at ClinicalTrials.gov (NCT01733277), was approved by the ethics committee IRB Services (Institutional Review Board Services), Aurora, ON, Canada. Written informed consent was obtained from all participants.

### Study visit: clinical, biological and X-ray outcomes

The clinical characteristics included demographic data, the Western Ontario and McMasters Universities Osteoarthritis Index (WOMAC) questionnaire [12] and the VAS for global knee pain (0 mm = no pain, 100 mm = most severe pain) within the last week, and the PainDETECT questionnaire completed at the study visit. Blood tests for sedimentation rate and C-reactive protein (CRP) were done. Knee X-rays taken within the last 12 months were used to verify that the subject satisfied radiological criteria of inclusion (KL grades 2 and 3).

### MRI acquisition and determination of structural changes

MRI of the target knee was performed no more than 21 days after the study visit. The MRI acquisitions were performed on Philips Achieva 3 T units (Philips HealthCare, Markham, ON, Canada). The MRI examination comprised the following sequences: sagittal proton density-weighted fast spin-echo sequence with fat suppression (PD-FSE) (TR/TE, 3,550/25 ms; flip angle, 90 degrees; slice thickness/gap, 3/0 mm; excitation number, 1; matrix size, 348 × 348 px; field of view, 140 mm; resolution, 0.398/0.398 mm; receiver bandwidth, 192 Hz/pixel; phase direction, S/I); sagittal three-dimensional intermediate-weighted fast spin-echo sequence with fat suppression (VISTA-SPAIR) (TR/TE, 1,500/30 ms; flip angle, 90 degrees; slice thickness/gap, 0.6/0 mm; excitation number, 1; matrix size, 232 × 232 px; field of view, 140 mm; resolution, 0.3125/0.3125 mm; receiver bandwidth, 380 Hz/pixel; phase direction, A/P); and axial T1-weighted gradient-echo sequence non-fat suppressed (In-Out Phase) (TR/TE, 450/3.45 to 4.60 ms; slice thickness/gap, 3.0/0 mm; excitation number, 1; matrix size, 256 × 256 px; field of view, 180 mm; resolution, 0.25/0.25 mm; phase direction, L/R).

The cartilage volume was measured using the VISTA-SPAIR sequence and values determined with ArthroLab’s fully automated quantitative MRI (qMRI) system (ArthroLab Inc, Montreal, QC, Canada) as described [13]. Meniscal tears were assessed with a fluid-sensitive sequence (PD-FSE) as recommended [14,15] and scored as absence (−) or presence (+) of a tear detected in any of the three segments (anterior horn, body, posterior horn) of the meniscus. Meniscal extrusion was assessed using the VISTA-SPAIR sequence, which allows for a precise evaluation of the extent of the extrusion, and scored as absence (−) or presence (+) of partial or complete extrusion detected in any of the three segments of the meniscus as described [14,16]. Presence of bone marrow lesions (BMLs) was assessed with the PD-FSE sequence and evaluated as described [17]. The synovial membrane thickness (mm) was measured with the In-Out Phase sequence and measured in four subregions (medial and lateral articular recess and medial and lateral border of the suprapatellar bursa) as described [17,18]. Of note, the measurement of the synovial membrane thickness according to this method relies on the presence of synovial fluid, as assessed with the PS-FSE sequence, to localize the membrane in the different regions of interest. Thus, absence of synovial fluid that occurs, especially for the medial suprapatellar bursa, accounts for some missing values. The synovial effusion volume was assessed in its entirety using ArthroLab’s fully automated qMRI system as described [19]. The evaluation of the structural changes was done under blinded conditions of patient identification and clinical data.

### Statistical analysis

This exploratory study aimed to correlate the knee OA structural changes as assessed by quantitative and semi-quantitative MRI scoring systems (cartilage volume, meniscal extrusion, BMLs, synovitis, and synovial effusion) with the presence of NP, by comparing the three study groups stratified according to PainDETECT scores (≤12, 13 to 18, and ≥19). Comparison of the demographic characteristics of the three study groups was carried out using the Kruskal-Wallis test for continuous variables and the chi-square test (or Fisher’s exact test) for categorical variables. Comparison of the clinical and MRI characteristics was carried out using the Jonckheere-Terpstra trend test for continuous variables and the Cochran-Armitage trend test for categorical variables. A *P* value ≤0.05 was considered statistically significant. All statistical analyses were done using SAS software, version 9.3 (SAS Institute Inc., Cary, NC, USA).

## Results

### Demographics

The demographic characteristics of the three groups did not differ (Table 1). No statistical difference was found between PainDETECT groups with regard to the KL scores (Table 1). None of the patients received treatment for NP such as tricyclic antidepressants (TCAs), selective serotonin and norepinephrine reuptake inhibitors (SSRIs and SNRIs, respectively) or anticonvulsants (for example calcium channel α2δ ligands).

**Table 1:** **Demographic and clinical characteristics of patients based on PainDETECT score**

	**PainDETECT ≤12 n = 25**	**PainDETECT 13-18 n = 9**	**PainDETECT ≥19 n = 16**	***P*** **value**
**Demographic and clinical**				
Age, years	66 ± 9	63 ± 9	67 ± 7	0.392*
Female, n (%)	18 (72%)	5 (56%)	10 (63%)	0.629‡
Weight, kg	82.5 ± 14.1	81.0 ± 10.6	87.0 ± 18.9	0.587*
OA duration, years	7.7 ± 8.1	7.4 ± 6.6	8.1 ± 4.6	0.528*
BMI, kg/m^2^	31 ± 6	29.9 ± 3	33 ± 6	0.558*
NSAID users, n (%)	3 (12%)	0 (0%)	5 (31%)	0.114¶
PainDETECT	8.1 ± 2.6	15.6 ± 1.1	24.2 ± 4.2	**<0.001♦**
WOMAC				
Pain (0–100)	43 ± 17	50 ± 13	65 ± 14	**<0.001♦**
Function (0–100)	45 ± 23	52 ± 15	68 ± 14	**<0.001♦**
Stiffness (0–100)	51 ± 24	49 ± 18	69 ± 16	**0.007♦**
Total (0–100)	45 ± 21	51 ± 14	68 ± 13	**<0.001♦**
VAS pain (0–100 mm)	62 ± 14	62 ± 13	69 ± 13	**0.023♦**
**Kellgren-Lawrence score**				0.068†§
2	14 (56%)	5 (56%)	5 (31%)	
3	11 (44%)	4 (44%)	11 (69%)	
**Inflammatory biomarkers**				
Sedimentation rate, mm	12.4 ± 9.0	12.6 ± 5.5	15.2 ± 20.8	0.406♦
CRP, mg/l	4.3 ± 2.9	6.8 ± 9.1	5.0 ± 4.7	0.432♦

Results are shown as mean ± standard deviation (SD) unless otherwise indicated. *P* values were assessed using the *Kruskal-Wallis test, the ‡chi-square test, the ¶Fisher’s exact test, the **♦**Jonckheere-Terpstra test for trend, or the †Cochran-Armitage trend test; §Includes both Kellgren-Lawrence scores. n, number of participants; OA, osteoarthritis; BMI, body mass index; NSAID, non-steroidal anti-inflammatory drug; WOMAC, Western Ontario and McMaster Universities Osteoarthritis Index (each subscale, 100 = worst score; total scale, 100 = worst score); VAS, visual analog scale (0 mm = no pain, 100 mm = most severe pain); CRP, C-reactive protein.

### WOMAC, VAS, and PainDETECT pain scores

A significant association was found between the WOMAC pain (*P* <0.001), function (*P* <0.001), stiffness (*P* = 0.007) and total (*P* <0.001) scores and higher VAS pain (*P* = 0.023) and PainDETECT scores (Table 1).

### Inflammatory biomarkers

No association was found between the sedimentation rate, the CRP values, and the PainDETECT scores (Table 1).

### Knee structural changes assessed by MRI

#### Cartilage

No association was found between the cartilage volume and the PainDETECT scores (Table 2).

**Table 2:** **Knee structural changes assessed by MRI in patients based on PainDETECT score**

	**OA/NP- PainDETECT ≤12 n = 25**	**OA/NP+ PainDETECT 13-18 n = 9**	**OA/NP+ PainDETECT ≥19 n = 16**	***P*** **value♦**
**MRI cartilage volume (mm** ^**3**^ **)**				
Global knee	12,439 ± 2,451	12,181 ± 3,894	12,280 ± 2,414	0.347
Femur	8,872 ± 1,799	8,998 ± 2,769	8,784 ± 1,950	0.381
Condyle	5,873 ± 1,220	5,678 ± 1,784	5,825 ± 1,552	0.288
Plateau	3,567 ± 907	3,183 ± 1,467	3,497 ± 727	0.395
Medial compartment	6,232 ± 1,258	5,919 ± 2,098	6,101 ± 1,473	0.333
Femur	4,594 ± 884	4,602 ± 1,451	4,474 ± 1,103	0.288
Condyle	3,094 ± 541	2,878 ± 912	3,043 ± 915	0.172
Plateau	1,639 ± 516	1,317 ± 791	1,626 ± 524	0.424
Lateral compartment	6,207 ± 1,404	6,207 ± 1,404	6,180 ± 1,476	0.300
Femur	4,278 ± 1,030	4,397 ± 1,415	4,309 ± 1,002	0.445
Condyle	2,779 ± 768	2,800 ± 1,019	2,782 ± 798	0.496
Plateau	1,928 ± 595	1,865 ± 766	1,870 ± 738	0.720
**Meniscal extrusion (presence)**				
Medial compartment	7 (28%)	3 (33%)	11 (69%)	**0.006**†
Lateral compartment	1 (4%)	1 (11%)	4 (25%)	**0.023**†
**Meniscal tears (presence)**				
Medial compartment	14 (56%)	5 (56%)	10 (63%)	0.347†
Lateral compartment	3 (12%)	1 (11%)	7 (44%)	**0.011**†
**Bone marrow lesions (presence)**				
Medial compartment	13 (54%)^a^	8 (89%)	10 (63%)	0.246†
Condyle	4 (17%)^a^	1 (11%)	3 (19%)	0.447†
Plateau	12 (50%)^a^	7 (78%)	10 (63%)	0.186†
Lateral compartment	9 (38%)^a^	5 (56%)	7 (44%)	0.321†
Condyle	1 (4%)^a^	0 (0%)	1 (6%)	0.395†
Plateau	8 (33%)^a^	5 (56%)	10 (63%)	**0.032**†
**Synovial membrane thickness (mm)**				
Global	2.23 ± 0.66	2.32 ± 0.51	2.13 ± 0.52	0.427
Medial compartment	2.27 ± 1.28^b^	2.14 ± 0.58	1.97 ± 0.42^c^	0.390
Medial recess	2.02 ± 0.96^d^	1.91 ± 0.62	1.93 ± 0.82^c^	0.399
Medial suprapatellar bursa	2.89 ± 1.57^e^	2.73 ± 1.00^f^	2.13 ± 0.68^e^	0.111
Lateral compartment	2.21 ± 0.75	2.47 ± 0.68	2.19 ± 0.73	0.402
Lateral recess	1.90 ± 0.64^a^	2.07 ± 0.76	2.15 ± 0.55^c^	**0.027**
Lateral suprapatellar bursa	2.64 ± 1.42^b^	2.96 ± 1.15^g^	2.26 ± 1.18	0.228
**Synovial effusion (ml)**	11.22 ± 7.74	14.40 ± 10.3	12.40 ± 6.40	0.202

Results are shown as mean ± standard deviation (SD) unless otherwise indicated. *P* values were assessed using the **♦**Jonckheere-Terpstra test for trend, and the †Cochran-Armitage trend test. ^a^, n = 24; ^b^, n = 22; ^c^, n = 15; ^d^, n = 21; ^e^, n = 12; ^f^, n = 6; ^g^, n = 8. MRI, magnetic resonance imaging; OA, osteoarthritis; NP, neuropathic pain; n, number of participants.

#### Meniscus

The presence of meniscal extrusion in both medial (*P* = 0.006) and lateral (*P* = 0.023) compartments was found to be significantly associated with increasing PainDETECT scores (Table 2). The presence of meniscal tears in the lateral compartment (*P* = 0.011) was also significantly associated with PainDETECT scores (Table 2).

#### Bone marrow lesions

Whereas no association between the presence of BMLs and the PainDETECT scores was found for the medial compartment, the association was significant in the lateral plateau (*P* = 0.032, Table 2).

#### Synovial membrane thickness/synovial effusion

No association was found between the synovial membrane thickness or synovial effusion size and the PainDETECT scores, except for the synovial membrane thickness in the lateral recess (*P* = 0.027) (Table 2).

## Discussion

The aim of this cross-sectional pilot study was to explore in symptomatic knee OA patients the relationship between structural changes assessed by MRI and the presence of NP.

In the studied population of symptomatic knee OA patients with moderate to severe pain level, data first indicate a positive relationship between the level of OA symptoms and NP, in which the WOMAC scores as well as the VAS pain score were associated with the PainDETECT scores. Importantly, data also showed a greater likelihood of NP in patients with meniscal extrusion and lateral meniscal tears. These thus suggest that knee OA patients with a neuropathic component of pain have more severe symptoms, which appear related to meniscal lesions, more specifically extrusion, and are reflected, to a certain extent, by a trend toward a greater consumption of non-steroidal anti-inflammatory drugs (NSAIDs).

A higher WOMAC pain score in OA patients with a neuropathic component of pain has been previously reported [4] and could possibly explain the greater level of central sensitization that may have occurred. There was, however, no evidence in the present study, based on the current MRI findings, of an association with a more severe disease, which is in agreement with the previous report [4]. Results of this study also concur with the report of Murphy *et al*. [20] to the effect that the level of centrally mediated symptoms was likely independently associated with the pain severity. One cannot exclude the fact that nociception may have also played a role in the genesis of local pain and peripheral hyperalgia in the OA knee joint as well as central sensitization related to prolonged neuronal discharges [21]. The combination of peripheral and central pain mechanisms may possibly explain, at least in part, the discrepancy reported between the severity of OA changes and the pain intensity.

The mechanisms underlying NP-like symptoms in OA are still poorly understood. OA pain likely includes both nociceptive and neuropathic components. It has been suggested that the local damage to innervation as well as other joint structures may cause damage to peripheral nerves [3]. In this context, the association found between the presence of NP and BMLs and synovitis (synovial membrane thickness) in the lateral compartment is also very interesting. Although BMLs have been associated with knee pain [22-24], no report so far has related those changes with NP. A definite study with a larger number of patients would provide a better understanding of the current finding as well as its true meaning. A similar comment applies to the observation regarding the increase in synovial membrane thickness in the lateral compartment, as it is believed that synovial inflammation could sensitize peripheral nociceptors [25]. Therefore, it would seem appropriate and logical to pursue this avenue of research in the future.

The association of knee pain with meniscal extrusion assessed by MRI in OA patients was recently reported [26]. A few possible explanations for the relationship between meniscal extrusion and knee pain are as follows. First, the association between meniscal extrusion and BMLs, a known source of pain [26], could possibly be due to the loss of mechanical protection provided by the meniscus. Second, it could be, as recently reported [27], that the increased vascular penetration and sensory nerve densities in the OA medial meniscus suggest a potential role of meniscal sensory nerve growth in knee OA pain, and could have contributed to the neuropathic component of the pain. Another possible explanation may be the mechanical stretching of the joint capsule, a richly innervated tissue, by the bulging meniscus. Our finding of an association between NP and lateral meniscal tear is somewhat unexpected as literature indicates that meniscal tears are not usually associated with symptoms [28]. Perhaps this finding is due to the role of possible confounding factors that will have to be further explored in a comprehensive study with a larger number of patients.

It is noteworthy that very few data exist regarding the relationship between meniscal lesions and NP in knee OA and this is a promising field of future research. The present study is particularly interesting as it is the first to report a clear association of meniscal lesions, more specifically extrusion, with NP. The finding of the association between the presence of meniscal extrusion and the PainDETECT scores makes this structural alteration a definite marker of NP. This finding is clinically relevant for various reasons. First, it argues for a pathophysiological relationship between NP and meniscal extrusion in knee OA and supports the examination for meniscal extrusion in knee OA patients with NP. This suggests that in daily practice, the predominance of a neuropathic component in such patients should encourage physicians to consider the use of MRI to establish a proper diagnosis. Second, a diagnosis of meniscal extrusion may help to identify knee OA patients who are more susceptible to benefit from a treatment aimed at controlling their symptoms more specifically. There is hope that this ‘personalized therapeutic management’ would avoid the prolonged use of anti-inflammatory drugs or even narcotic analgesics, preventing potential side effects, and that the patients would have a better response to treatment. Future research might explore whether managing meniscal extrusion, for instance with arthroscopic meniscal repair or resection, in such patients with NP would be beneficial.

This pilot study has limitations, the first being that it was an observational study and not a randomized controlled trial, the second being the arbitrary determination of the sample and, third, the diagnosis of NP was based solely on the PainDETECT questionnaire. However, as no gold standard test for NP diagnosis in OA is yet recognized, this questionnaire was used, as in many previous OA studies [4,29,30], since it provides the greatest level of confidence. The relatively small sample size may also be a limiting factor. Nonetheless, this MRI study provides strong insight into the relationship between NP and meniscal lesions in knee OA, especially in patients with higher PainDETECT scores, hence with likely NP.

## Conclusions

In summary, the findings of this study show that in knee OA patients, meniscal lesions are a definite major risk factor for NP. BMLs and synovitis also seem to be, to a certain extent, associated with NP; however, a definite study is needed to fully address the question, as they may be contributing factors in association with others.

## Abbreviations

BMLs: bone marrow lesions
CRP: C-reactive protein
KL: Kellgren-Lawrence
MRI: magnetic resonance imaging
NP: neuropathic pain
NSAIDs: non-steroidal anti-inflammatory drugs
OA: osteoarthritis
qMRI: quantitative MRI
SNRIs: selective norepinephrine reuptake inhibitors
SSRIs: selective serotonin reuptake inhibitors
TCAs: tricyclic antidepressants
VAS: visual analog scale
WOMAC: Western Ontario and McMasters Universities Osteoarthritis Index
